# Editor’s note: Disability in older people during wars: the Ukrainian war

**DOI:** 10.1007/s40520-024-02907-3

**Published:** 2024-12-27

**Authors:** Nicola Veronese

**Affiliations:** Saint Camillus International University of Health Sciences, Via di Sant’Alessandro 8, Roma, 00131 Italy

I personally invited the Ukrainian health experts to write a work about geriatric issues during the Russian invasion. Briefly, in this work, the authors examine disability trends among older adults due to non-communicable diseases (NCDs) between 2013 and 2023, with a particular focus on the drastic shifts observed during the ongoing Russian invasion [1]. The findings underscore the intersection of aging, health vulnerabilities, and the catastrophic impact of conflict on healthcare and well-being, offering crucial insights into a global issue of older populations at risk in conflict zones [1].

NCDs—like cardiovascular diseases, cancer, cerebrovascular diseases, and musculoskeletal conditions—were leading contributors to disability among Ukraine’s elderly even before the war. However, the war exacerbated these challenges, triggering an almost threefold increase in disability rates among older adults by 2023 compared to pre-war levels [1]. This dramatic surge in disability underscores the compound effect of war on a population already burdened by age-related health issues and limited healthcare resources [1]. 

In my opinion, this study highlights the grim reality for older Ukrainians in frontline regions, such as Zaporizhzhia and Kharkiv. These areas have witnessed a significant uptick in disabilities due to cardiovascular diseases, cancer, cerebrovascular diseases, and musculoskeletal issues, all aggravated by the relentless stresses of war. For instance, cardiovascular disease—a major cause of disability before the war—saw further escalation in affected numbers, likely due to stress, disrupted treatment, and diminished healthcare access [1]. 

Older people in frontline regions also endure heightened risks of psychiatric disorders, mobility impairments, and physical injuries from wartime violence. The prevalence of osteoarthritis-related disabilities surged, a reflection of both the physical strain from displacement and the limited access to timely medical intervention. For those able to relocate, cities with high concentrations of displaced persons, such as Dnipro and Odesa, face overcrowded healthcare systems, compounding the difficulty in accessing necessary treatments.

This nice study adds to the growing body of evidence indicating that prolonged exposure to conflict worsens NCDs. The constant stress and fear of violence, coupled with displacement and economic instability, accelerate health deterioration among older people other than factors widely known as accelerating the risk of disability [2]. This is particularly evident in conditions like coronary artery disease and cerebrovascular diseases, which are sensitive to stress-related exacerbations. Furthermore, the study draws parallels with past conflicts where increased rates of cardiovascular issues and mental health disorders were observed among war-affected populations, as seen in other studies of Syrian conflict survivors [1]. 

In this regard, it is important to remember that the surge in older disability from conditions like coronary artery disease and cancer is compounded by reduced primary preventive care [3]. Disruption to healthcare services leaves NCDs unmanaged, turning what might have been manageable conditions into permanent disabilities. For older individuals, preventive care such as regular screenings, medication adherence, and check-ups are vital; however, war-induced health system breakdowns lead to a lack of continuity in such care, creating a generational health crisis that will likely have lingering effects long after the conflict subsides.

In conclusion, this research shows, one time again, the need to consider older people among those more involved during wars. As Ukraine faces unprecedented healthcare challenges, the need for targeted, age-sensitive interventions is more pressing than ever. This study offers not only a lens into the humanitarian cost of the war on Ukraine’s older people but also a call to action for prioritizing healthcare for vulnerable populations in global conflict zones hoping in a quick and peaceful solution of this conflict.
